# Draft Genome Sequence of Lactiplantibacillus plantarum Strain ISO1, a Potential Probiotic Bacterium Isolated from the Milk of South African Saanen Goats

**DOI:** 10.1128/mra.01245-22

**Published:** 2023-01-18

**Authors:** Goitsemang Makete, Tshifhiwa Paris Mamphogoro

**Affiliations:** a Gastro-Intestinal Microbiology and Biotechnology Unit, Agricultural Research Council-Animal Production, Irene, Pretoria, South Africa; University of Maryland School of Medicine

## Abstract

Lactiplantibacillus plantarum strain ISO1, a potential probiotic with wide food and biotechnological applications, was isolated from the raw milk of South African Saanen goats. Here, we report the draft genome sequence of this bacterium. Sequencing yielded a 3.23-Mb genome and 2,967 protein-coding sequences.

## ANNOUNCEMENT

Lactiplantibacillus plantarum previously known as Lactobacillus plantarum is a Gram-positive rod shaped, nonmotile, nonspore, catalase-negative and facultative anaerobe species often reliable as bacteriocin producer and a promising probiotic species with wide application in the food and health industry (1, 2). It was found that *L. plantarum* can survive the bile and acidic environment of the colon epithelium (3). In addition, many strains of this bacterial species produce antimicrobials that prevent the growth of pathogenic microorganisms.

In this study, we sequenced the whole genome of *Lactiplantibacillus plantarum* strain ISO1, isolated from goats milk. A total of 40 raw goat milk samples were collected from the Small-Stock Division of the Agricultural Research Council, Animal Production (Irene, South Africa). For isolation and purification, 1 mL of each milk sample was aseptically added to 9 mL of sterile saline solution (0.85% [wt/vol] NaCl). The supernatant was serially diluted, plated onto De Man, Rogosa, and Sharpe (MRS) agar supplemented with 0.05 g/L cysteine-HCl (MRS-cysHCl), and incubated at 37°C for a period of 48 h under microaerophilic conditions. After incubation, distinct colonies were streaked onto MRS-cysHCl agar. The pure strain was subcultured in MRS-cysHCl broth, incubated at 37°C for 48 h, and stored in 10% (vol/vol) glycerol at −20°C (4). Genomic DNA was extracted using the Quick-DNA fungal/bacterial miniprep kit (Zymo Research, Irvine, CA) and amplified using 16S rRNA universal primers (1541R and 27F) (5). The amplicon was sequenced, a similarity analysis was performed using the BLAST server, and the sequence was submitted to GenBank. Revived *L*. *plantarum* ISO1 cells were then subcultured on MRS-cysHCl agar, and the biomass was used for DNA extraction.

Genomic DNA of strain ISO1 was sequenced on the Illumina NextSeq platform at Inqaba Biotechnical Industries (Pty) Ltd. (Pretoria, South Africa). The NEBNext Ultra II FS DNA library prep kit (New England Biolabs, Ipswich, MA) was used for DNA library preparation. The libraries were then sequenced on the Illumina NextSeq 550 platform, and a total of 2,227,139 reads generated from each sample (2 × 150-bp paired-end reads) were visualized using the KBase platform (6). The quality of the reads was evaluated using FastQC v0.11.5 (7), while low-quality reads and sequence adaptors were removed using Trimmomatic v0.36 (8). The genome assembly was performed using SPAdes v3.13.0 (9) with default parameters, yielding 36 contigs with a coverage of 300×. The assembly yielded a genome sequence 3,233,046 bp long, with a G+C content of 44.5%. The contigs had *N*_50_ and *L*_50_ values of 358,672 bp and 4, respectively. Gene annotation was performed using the Rapid Annotations using Subsystems Technology toolkit (RASTtk) v1.073, as well as the NCBI Prokaryotic Genome Annotation Pipeline (PGAP) v6.3 (10, 11), which identified 2,967 protein-coding genes, 62 RNA genes, 3 rRNA genes, 55 tRNA genes, and 44 pseudogenes.

The reported draft genome sequence of ISO1 will help us improve strain-specific fingerprinting, which is crucial for the registration and protection of commercial probiotic products. Furthermore, this draft genome sequence will provide a platform for elucidating various probiotic mechanisms and their regulation.

### Data availability.

This whole-genome shotgun project has been deposited at DDBJ/ENA/GenBank under the accession number JAPJDR000000000. The version described in this paper is the first version. The SRA accession number is SRR22299852, the BioProject accession number is PRJNA896361, and the BioSample accession number is SAMN31723195. The 16S rRNA gene amplicon sequence is available under the GenBank accession number OP824881.
